# Reductions in Inpatient Mortality following Interventions to Improve Emergency Hospital Care in Freetown, Sierra Leone

**DOI:** 10.1371/journal.pone.0041458

**Published:** 2012-09-19

**Authors:** Matthew Clark, Emily Spry, Kisito Daoh, David Baion, Jolene Skordis-Worrall

**Affiliations:** 1 Imperial College Healthcare NHS Trust, Praed Street, London W2 1NY; 2 Welbodi Partnership, Unit 14, Garrick Industrial Centre, London NW9 6AQ; 3 Minstry of Health and Sanitation, 4th floor Youyi Building, Freetown, Sierra Leone; 4 Ola During Children's Hospital, Fourah Bay Road, Freetown, Sierra Leone; 5 UCL Centre for International Health & Development, Institute of Child Health, 30 Guilford Street, London, WC1N 1EH; University of Maryland, United States of America

## Abstract

**Background:**

The demand for high quality hospital care for children in low resource countries is not being met. This paper describes a number of strategies to improve emergency care at a children's hospital and evaluates the impact of these on inpatient mortality. In addition, the cost-effectiveness of improving emergency care is estimated.

**Methods and Findings:**

A team of local and international staff developed a plan to improve emergency care for children arriving at The Ola During Children's Hospital, Freetown, Sierra Leone. Following focus group discussions, five priority areas were identified to improve emergency care; staff training, hospital layout, staff allocation, medical equipment, and medical record keeping. A team of international volunteers worked with local staff for six months to design and implement improvements in these five priority areas. The improvements were evaluated collectively rather than individually. Before the intervention, the inpatient mortality rate was 12.4%. After the intervention this improved to 5.9%. The relative risk of dying was 47% (95% CI 0.369–0.607) lower after the intervention. The estimated number of lives saved in the first two months after the intervention was 103. The total cost of the intervention was USD 29 714, the estimated cost per death averted was USD 148. There are two main limitation of the study. Firstly, the brevity of the study and secondly, the assumed homogeneity of the clinical cases that presented to the hospital before and after the intervention.

**Conclusions:**

This study demonstarted a signficant reductuion in inpatient mortality rate after an intervention to improve emergency hospital care If the findings of this paper could be reproduced in a larger more rigorous study, improving the quality of care in hospitals would be a very cost effective strategy to save children's lives in low resource settings.

## Introduction

Approximately eight million children under the age of five die every year. The vast majority of these children live in the low-income countries and suffer from easily preventable or treatable disease [1]. Many deaths being averted by primary prevention strategies and community-based treatment. However, it is estimated that up to 45% of children will require hospitalization at some point in their lives [2], [3], [4].

The high demand for hospital care for children in low resource countries is not being met with adequate provision of quality hospitals [5]. Literature on the quality of hospitals in low resource countries, while extremely limited, suggests that those children who do access a hospital receive poor care. A survey of 13 district hospitals in Kenya identified widespread deficiencies in case management [6]. In a survey of 21 hospitals in low-income countries, there were significant deficiencies in the case management of 76% of the children treated. That study suggested specific areas in need of improvement included triage, emergency care, assessment, inpatient treatment and monitoring [5]. Poor case management of children leads to inpatient mortality rates as high as 15–18% [6] [2]. The consequences of high inpatient mortality rates extend far beyond the immediate case fatalities. Poorly functioning hospitals can cause surrounding patients to delay attending hospital until the child is *in extremis* or to spurn conventional medicine altogether in pursuit of traditional remedies or private treatment, thus further increasing the risk of avoidable mortality and morbidity [7] [8].

Published literature on interventions to improve the quality of clinical care in hospitals in low-income countries is scarce. In Guinea-Bissau, supervision and incentives for healthcare workers to adhere to standardized treatment protocols reduced malaria mortality rates by up to 50% [11]. Introducing oxygen concentrators and pulse oximeters in hospitals in Papua New Guinea reduced the risk of dying of pneumonia in hospital by 35% [12]. A recent study in Kenya reports improvements in “process-of-care measures” after a multifaceted intervention to improve care [13].

This paper describes a number of strategies to improve emergency care at a government children's hospital in Freetown, Sierra Leone and evaluates the impact of these strategies on inpatient mortality. In addition to impact, the cost-effectiveness of improving emergency care is also estimated.

## Methods

### Setting

The Ola During Children's Hospital (ODCH) is located in the densely populated and impoverished eastern part of Freetown, Sierra Leone. The hospital was established as a tertiary referral hospital, but in reality ODCH serves as a combined primary and secondary health care facility for children of Freetown. Referrals from hospitals outside of Freetown to ODCH are rare and the hospital lacks many of the facilities normally associated with referral hospitals in the region. ODCH is a government hospital that receives additional support from two international non-government organisations.

In September 2009, ODCH had one hundred and eight inpatient beds divided across three wards, a very basic intensive care unit with one oxygen concentrator, a therapeutic feeding centre and an outpatient department. There were basic laboratory facilities but no radiology department. The hospital was staffed by one specialist paediatrician, six medial officers, twenty registered nurses and a larger team of nursing assistants. No formal triage or emergency departments were in use and patients would be seen in the order they arrived at the hospital, with nurses or registration clerks occasionally identifying and prioritising particularly sick patients.

In 2008 ODCH admitted approximately 300 patients a month on average and the monthly inpatient mortality rate ranged from 13.5%–22.4%. In August 2009 ODCH changed from full cost-recovery to a flat-rate user fee system. Under the flat-rate system, all patients pay a Le 15 000 ($5) registration fee. This fee covers an outpatient assessment, admission if required and a follow up appointment within two weeks. If patients are admitted, all medication and consumables are available at no extra charge, while outpatient medications are sold to parents at the cost-recovery pharmacy. In April 2010 (after the intervention period studied), the flat rare user fee was superseded by a national policy to provide fee health care for all children under five. In September 2009, one month after the introduction of the flat-rate user fee, ODCH admitted approximately 600 children over the course of the month. In that month, the inpatient mortality rate was 13.2%, similar to the inpatient mortality rate in September 2008 (13.1%).

### Intervention: Improving emergency care

In response to the persistent high inpatient mortality rate and the increased number of patients, a team of local and international staff aimed to improve emergency care for children arriving at ODCH. As discussed above, the literature on how best to tackle high inpatient mortality rates is limited and suggests that training, staff supervision and oxygen concentrators are effective [11] [12] [13]. The unique feature of this intervention is the degree of local staff participation in the design. The emphasis on local participation staff was based on anecdotal experience rather than any previously published research.

**Figure 1:**
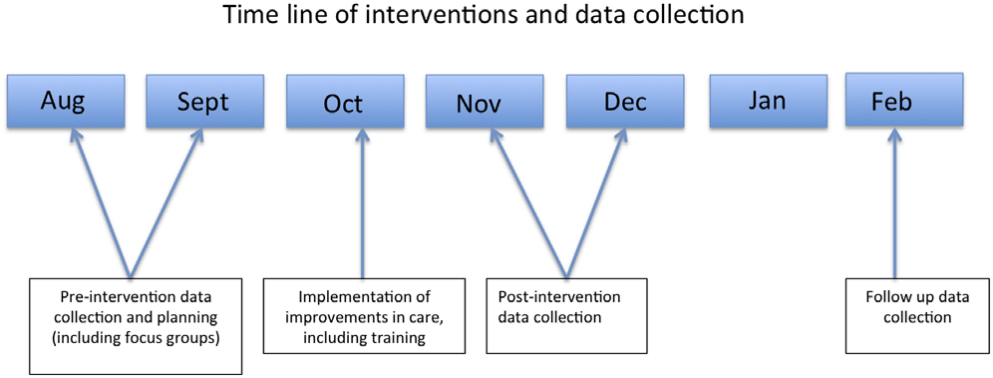
time frame for improving emergency care.

Through focus group discussions with hospital staff (including doctors, nurses and hospital management), five priority areas were identified to improve emergency care i.e.; staff training, hospital layout, staff allocation, medical equipment, and medical record keeping. These proposals were presented to and approved by The Ministry of Health and Sanitation before implementation. A team of three international doctors, worked with local staff for six months to design and implement the changes outlined below. Changes in care practise were introduced incrementally over a one-month period in October 2009. By November 2009 all the systems described below were in place. The hospital management committee and The Sierra Leone Ministry of Health and Sanitation approved all plans. *Figure 1* summarises the time frame for improving emergency care.

*Training:* To address the training needs of staff at ODCH, a four day emergency triage and treatment course for nurses was run by a team of four visiting nurse from the UK in conjunction with the volunteer doctors. This course was based on The WHO Emergency Triage Assessment and Treatment (ETAT) course and adapted based on the local needs identified during the focus groups [10]. The training programme can be found in *Figure S1*. In addition to the formal teaching, every opportunity was taken by international staff to provide *ad hoc* training in both clinical and non-clinical skills. At no stage did the visiting doctors and nurses take over day-to-day clinical care, throughout the post intervention clinical care was delivered by local doctors and nurses.

*Hospital Layout:* Two major limitations with the layout of the hospital were identified. Firstly, there was no designated area within the hospital to triage patients. Secondly, critically ill patients were spread across the outpatient department, the intensive care unit and the wards. After morning rounds it was common for doctors not to review patients on the wards and intensive care until the next morning. To address these problems, two changes were made; Firstly, one ward was converted into a combined emergency room and intensive care unit. The proximity of the Emergency Room and intensive care unit, meant that the medical officer assigned to the emergency room was also more able to review patients in intensive care. Secondly, an area in the outpatient department was converted into a triage area.

*Medical Equipment*: The new triage area at the hospital was equipped with scales, thermometers and pulse oximeters. The new Emergency Room was equipped with three patient trolleys, emergency drugs and disposables, an oxygen concentrator, a pulse oximeter and a glucometer. The new intensive care unit had 18 beds (although when the hospital was busy it was not unusual to have more than one child to a bed). The intensive care unit was equipped with an additional two oxygen concentrators, pulse oximeters and glucometers, in addition to the existing supply of drugs and consumables.

*Staff allocation:* The hospital matron allocated a team of nursing aides to the triage areas from existing wards. The triage nurses were responsible for recording basic observations of pulse, respiratory rate and oxygen saturations. Based on the observations and clinical symptoms, children were triaged into three categories; emergency, priority and routine. Emergency patients were sent immediately to the Emergency Room for further assessment and treatment. The remaining patients were asked to sit on either a priority or routine bench in the outpatient department.

The emergency room was staffed by some of the most experienced nurses in the hospital who had all undergone the ETAT course. In addition, a medical officer was assigned to the emergency room 24 hours a day. If children remain critically ill after emergency treatment they were transferred to the intensive care unit adjacent to the emergency room for ongoing treatment.

*Medical record keeping:* Finally, a structured medical clerking pack was introduced. This meant that all children had single medical records that included space for nurses to record triage findings and a structured proforma for doctors to record their clinical findings. The clerking pack was based on that used by The Kenyan Paediatrics Association and has can be seen in *Figure S2*.

### Data collection

The primary outcome of interest was inpatient mortality rate. Data collected by the hospital medical records department was used to calculate inpatient mortality rate and the relative risk of dying before and after the interventions. There are some concerns about the quality of data collected by the hospital medical records department. There is a particular concern that the inpatient mortality rates are artificially low as children who die early in their admission do not always get fully registered with the hospital. With the advent of the clerking pack it became far more unusual for deaths to be missed in this way. Therefore, using the medical record's department data may conservatively estimate the efficacy of the intervention. The medical records department were unaware that their data would be used for this analysis, minimising the risk of sudden change in the way data was collected. It was also impossible to retrospectively acquire more accurate data with the resources available to the project, so it was decided to use official hospital records throughout to ensure consistency between the before and after measures of effect.

Patient characteristics in the pre and post intervention period were obtained from the hospitals database. Unfortunately, the database was incomplete and did not contain information on every patient. Therefore, a sample of 500 patients in the pre and post intervention period were extracted for analysis.

### Data analysis

The mortality rate during August and September 2009 was calculated for use as a baseline measure. Data from the month of October was excluded, as this was when the interventions were being introduced and there was a high level of input from the team of international doctors during this month. We then calculated the mortality rate in November and December 2009 as the post intervention measure. The relative risk of the dying in the post intervention period was calculated with 95% confidence intervals. We estimated the number of lives saved as the difference between the expected and actual number of deaths in the two months pre (August and September) and post implementation (November and December) period. It is expected this will give a conservative estimate of the number of deaths averted as trends over the last three years show a seasonal increase in mortality rate in October, November and December. (*Graph S1*) There is no obvious explanation for this seasonal trend.

### Cost and cost effectiveness

All costs were prospectively recorded in USD during the implementation of the intervention. Costs included fixed and variable costs, direct and indirect costs. Fixed costs included medical equipment, technical assistance and training of local staff. The value of medical equipment was calculated assuming straight-line depreciation from the purchase price over the manufactures expected lifespan. In addition, 10% shrinkage was incorporated into the costing model to allow for equipment malfunction, misuse and theft. The technical assistance and training course was delivered by volunteer doctors and nurses. While the direct costs of these volunteers are accounted for in the model, no attempt was made to calculate their lost earnings as it is assumed that future similar projects would be able to recruit volunteers on the same basis and that volunteers are able to use annual leave without incurring a loss of income.

Variable costs included consumables such as batteries and test strips for glucometers. Resource usage was calculated based on the actual number of children treated during the implementation month and the two subsequent months. Shrinkage of 20% was incorporated into the model for variable costs as these items were more likely to be misused.

The total costs of the intervention were estimated over three months, the one-month intervention period and the subsequent two months. The cost-effectiveness is calculated by dividing the estimated number of lives saved by the cost of the intervention. This is expected to provide a conservative estimate of the cost per life saved as we expect the benefits of the technical assistance and training course to extend by the two months after the intervention.

*Ethical approval:* No ethical approval was required for this manuscript.

## Findings

Patient characteristics in the pre and post intervention group are similar. The findings are summarised in *Table 1*.

**Table 1:** Patient characteristics in a random sample of 500 patients before and after the intervention took place.

	Pre	Post
Median age in months (+/−IQR)	24.5 (+/−12.4–48.7)	22.0 (+/−10.0–48.1)
Number male	274 (54.8)	276 (55.2)
Number female	226 (45.2)	224(44.8)
Number neonates (<28 days)	9 (1.8)	5 (1%)
Median duration of admission (+/−IQR)	4.0 (+/−3.0–6.0)	4.0 (+/−2.0–6.0)

The number of patients admitted by the hospital and the inpatient mortality rate is summarized in *graph S 2*.

The inpatient mortality rate was 12.4% in the pre-intervention period and 5.9% in the post intervention period. The relative risk of inpatient death was 47% lower than before the intervention. The expected number of deaths in the post intervention period was 195, the actual number of deaths was 92 so we estimate 103 deaths were averted. The mortality rate remained significantly reduced four months after the intervention. For brevity these estimates are summarised in *Table 2*.

**Table 2:** Summary of changes in mortality rate before, after and four months after the intervention.

	Pre	Post	4 months post
Number of cases	1204	1572	628
Number of deaths	149	92	46
Number of survivors	1055	1480	582
Mortality rate	12.38%	5.85%	7.32%
Relative risk of death post intervention	0.47		
95% Confidence interval	0.369–0.607		
Relative risk of death 4 mnths post	0.59		
95% CI	0.431–0.812		
Estimated numer of deaths averted	103		
Cost per death averted (USD)	147.58		

Including, relative risk of dying after the intervention, estimated number of lives saved after the intervention and costs per life saved.

The total cost of the intervention was USD 29 714, a break down of the costs is summarised in *Table 3*. The estimated cost per death averted is USD 148.

**Table 3:** Breakdown of costs of the intervention.

	Total cost (USD)	Cost for three months (USD)	Cost per life saved (USD)
**Fixed costs**			
***Equipment***			
Scales	62		
Pulse Oximeter (Nonin 9500 Onyx Finger Pulse Oximeter)	1294		
Oxygen Concentrator (Oxymat 3)	3963		
Patient Trolley	190		
Glucometer (Aviva Accucheck)	60		
Thermometers (Boots)	70		
Sub - total	5639	551	5
***Technical assistance***			79
Flights	2558		
Accomodation	4050		
Health Insurance	1004		
Visa	465		
Sub - total	8077	8077	79
***Training course costs 5 days costs***			
Refreshments:	300		
Stationary	50		
Training material	7		
Flights for inernational volunteers	2558		
Accomodation for international volunteers	1200		
Sub-total	4114	4114	40
**Variable costs**			23
Glucometer strips	620		
Batteries for pulse oximters	32		
Batteries for glucometer	12		
Sub-total	664	2390	23
**Total**	18494	15132	148

## Discussion

The results presented in this paper show that it is possible to significantly reduce short-term inpatient mortality by improving the quality of care at a cost of USD 148 per death averted.

The results must however be interpreted with caution, as this study has several limitations. The brevity of the study and the fluctuating number of inpatients over the previous year may confound our results. Ideally the monthly mortality rate over a 12 month period before and after the intervention should be used to provide more information about the sustainability of improvements in mortality rate and would more accurately allow for seasonal variation in mortality rates. The reason for the brief post intervention period was the introduction of free health care for child in Sierra Leone in April 2010 [14]. This policy shift doubled the number of inpatients and trebled the number of outpatients at The Ola During Children's Hospital. Such changes in the health systems context made it impossible to attribute changes in the mortality rate to the interventions to improve the quality of care described in this paper.

A further limitation of this study is the assumed homogeneity of the clinical cases that presented to the hospital before and after the intervention. It is possible that, as result of extra training, the clinical threshold for admitting children was reduced and this contributed to a reduction in inpatient mortality. However, it is reassuring that there was no difference in the duration of admission after the intervention. If it were that case, that post intervention children were less severely ill a reduced duration of admission would be expected. Furthermore, the concern about a lower threshold for admission is not supported by a sustained increase in the number of admissions. In any future trials in this area, it would be desirable to collect information about the clinical condition of children when they present to the hospital. It would also be useful to use some of the process outcomes such as adherence to WHO guidelines or time lag between arriving at the hospital and starting treatment.

The cost per death averted in this analysis compares well to the study in Papua New Guinea discussed above, where the cost was USD 1673 per death averted. In addition, this cost effectiveness compares favourably to other widely implemented public health measures including, insecticide treated mosquito nets ($438–2199), rota virus vaccine ($662) or the pneumococcal vaccine ($3200). [15] [16]xref> 17]. In summary, the intervention to improve emergency hospital care described in this paper is a comparatively cost effective way to save lives.

If the findings of this paper could be reproduced in a larger more vigorous study, improving the quality of care in hospitals would be a very cost effective strategy to save children's lives in low resource settings. With over half a million hospital beds in Sub-Saharan Africa, many of them providing sub standard care, the expansion of such programmes could save large numbers of lives and help accelerate progress towards Millennium Development Goal 4 [18] [5] [9].

## Supporting Information

Graph S1 Inpatinet mortality rate 2006–2008
(TIFF)

Graph S2 Number of inpatients and mortality rate August 2009–March 2010
(TIFF)

Figure S1 Medical clerking pack
(PDF)

Figure S2 Programme for triage and emergency training course.
(PDF)
