# Supplementary figures and images for: Reductions in Inpatient Mortality following Interventions to Improve Emergency Hospital Care in Freetown, Sierra Leone
Source: PLoS One. 2012 Sep 19;7(9):e41458. doi: 10.1371/journal.pone.0041458 (PMC3446969; doi:10.1371/journal.pone.0041458)

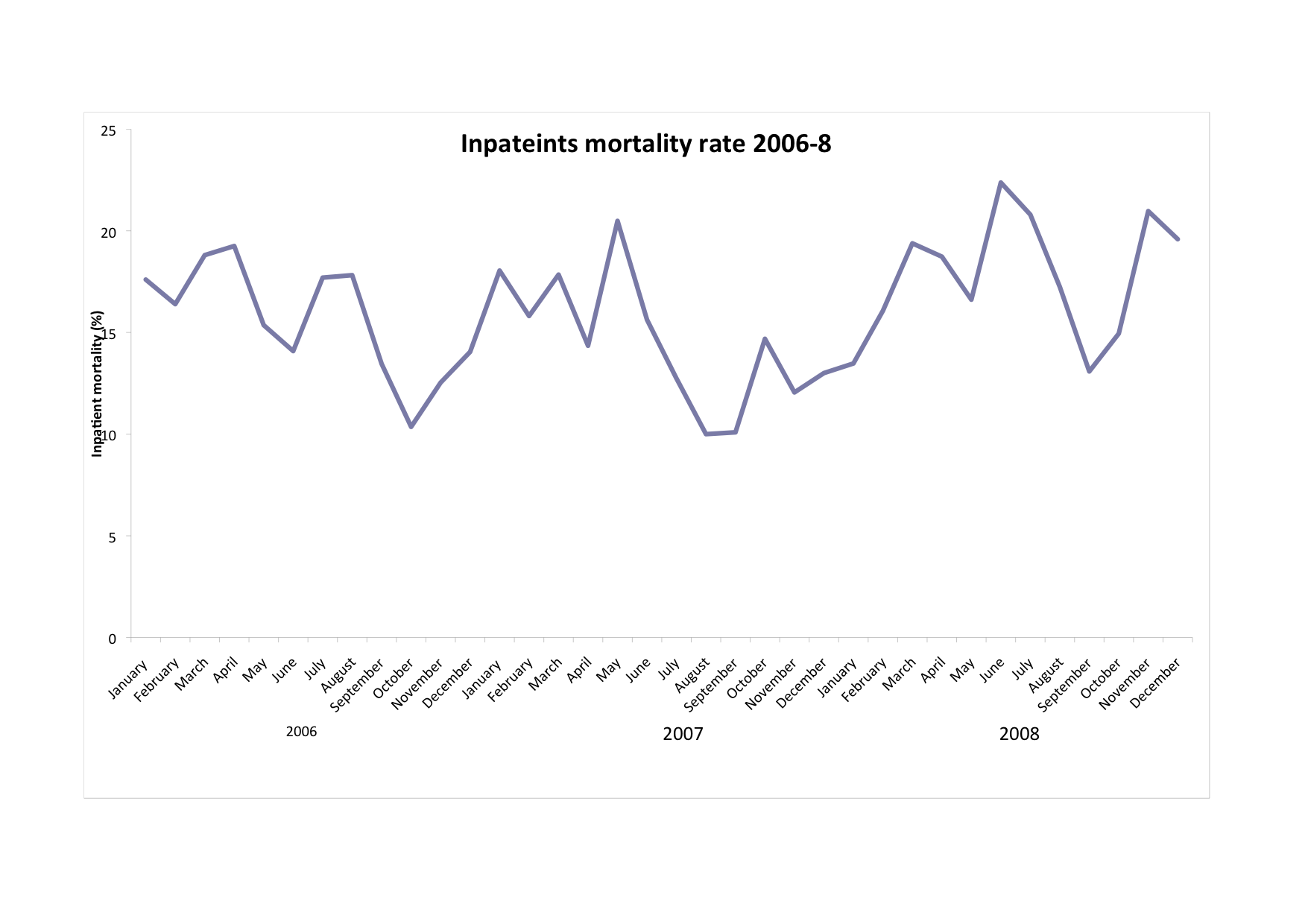

Supplement: Graph S1 — Inpatinet mortality rate 2006–2008 (TIFF) [file pone.0041458.s001.tif]

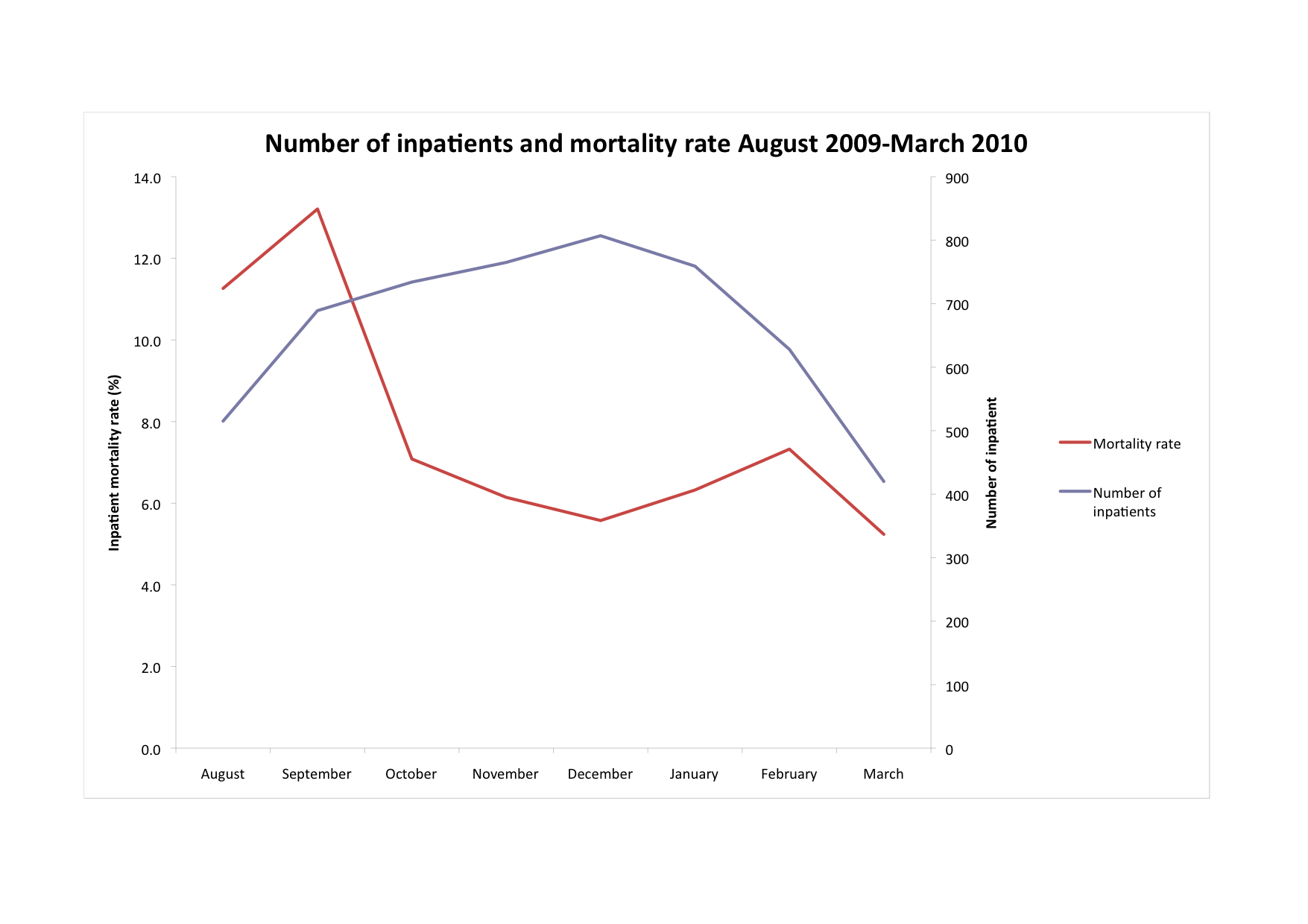

Supplement: Graph S2 — Number of inpatients and mortality rate August 2009–March 2010 (TIFF) [file pone.0041458.s002.tif]
