# Supplementary material for: Reductions in Inpatient Mortality following Interventions to Improve Emergency Hospital Care in Freetown, Sierra Leone
Source: PLoS One. 2012 Sep 19;7(9):e41458. doi: 10.1371/journal.pone.0041458 (PMC3446969; doi:10.1371/journal.pone.0041458)
Supplement: Figure S1 — Medical clerking pack (PDF) [file pone.0041458.s003.pdf]

Ola During Children’s Hospital - Nurses admission sheet

**Name**		**Sex**	Male/female
**Address**			
**Date of birth**		**Age**	

Date of Admission:

Weight: (Kg)		Height: (cm)		Weight for length:	
Temp: (C)		Pulse: (bpm)		RR:	
O2 Sats: (%)		Blood Glucose:		Hb:	

Emergency:	
Priority	
Queue	

Ola During Children’s Hospital - Doctors Clerking Sheets

Presenting Complaints

History			no. of days
Length of illness			
Fever	Y	N	
Difficulty Breathing	Y	N	
Wheeze	Y	N	
Cough	Y	N	
Diarrhoea (watery stools)	Y	N	
Diarrhoea (bloody stools)	Y	N	
Stools / 24 hours			
Difficulty Feeding	Y	N	
Vomiting	Y	N	
Vomiting/24 hours			
Vomiting everything	Y	N	
Convulsions	Y	N	
- intermittent	Y	N	
-persistent	Y	N	
**Birth History**			
Unattended delivery	Y	N	
Traditional Birth Attendant	Y	N	
Midwife present	Y	N	
Assisted delivery	Y	N	
C-Section	Y	N	

Examination

**A & B**	Stridor	Y	N	Resp. Exam	
	Central cyanosis	Y	N		
	Peripheral cyanosis	Y	N		
	Nasal Flaring	Y	N		
	Chest in-drawing	Y	N		
	Grunting	Y	N		
	Head nodding	Y	N		
	Wheeze	Y	N		
**C**	Pulse	weak		normal	strong
	Cap Refill (sec)	<2		2 -3	>3
	Sunken eyes	Y	N		
	Skin Pinch (sec.)	0		1	>1
	Pallor	0	+	+ +	+++
**D**	AVPU	A	V	P U	
	Can drink/breast feed?	Y	N		
	Bulging Fontanelle	Y	N		
	Stiff neck	Y	N		
**GEN**	Jaundice	0	+	++	+++
	Wasting	0	+	++	+++
	Oedema	Y	N		
	Oral Thrush	Y	N		

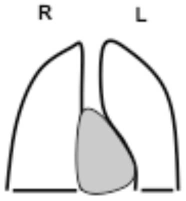

Other Examination:

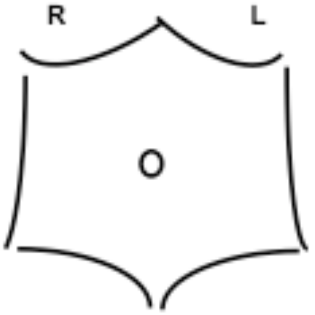

**Vaccination:**			
BCG & Oral Polio			
Pentavalent Vaccine	1	2	3
Yellow Fever & Measles			

**Significant PMH and treatment history:**		
Traditional medicine	Y	N
Pharmacy	Y	N
Arab Clinic	Y	N
Other treatment:		

**Primary diagnosis**					
**Pneumonia**	V. Servere	Severe	Non-severe		
**Diarrhoea**	Non-bloody	Bloody	**Dehydration**	Severe	Some
**HIV/AIDS**	+ve	-ve	unknown		
**TB**	Pulmonary	Extra-pulmonary			
**Malnutrition**	Kwash.	Marasm.	M. Kwash		
**Malaria**	Severe	lon - sever	**Anaemia**	Severe	Non - severe
**Meningitis**	Yes	No	**Neonatal sepsis**	Yes	No
**Birth asphyxia**	Yes	No	**Prematurity/ VLBW**	Yes	No
**Circulatory Shock**	Yes	No	**UTI**	Yes	No
**Other**					

**Secondary diagnosis**					
**Pneumonia**	V. Servere	Severe	Non-severe		
**Diarrhoea**	Non-bloody	Bloody	**Dehydration**	Severe	Some
**HIV/AIDS**	+ve	-ve	unknown		
**TB**	Pulmonary	Extra-pulmonary			
**Malnutrition**	Kwash.	Marasm.	M. Kwash		
**Malaria**	Severe	lon - sever	**Anaemia**	Severe	Non - severe
**Meningitis**	Yes	No	**Neonatal sepsis**	Yes	No
**Birth asphyxia**	Yes	No	**Prematurity/ VLBW**	Yes	No
**Circulatory Shock**	Yes	No	**UTI**	Yes	No
**Other**					

**Investigations**			
**Malaria parasites**	0 + ++ +++	**Hb**	
**Glucose**		**WCC**	
**HIV**	+ve -ve	**Glucose**	
**LP**		**Sickling**	
**Typhoid titre**		**Other**	
**Chest Xray**			

Management

Outpatient	Observation	Ward	ICU

Oxygen	
IV Fluids	
Blood products	

Date	Route	Medication	Frequency	Duration

Name and Grade	Signature	Time

Discharge Summary:

Outcome:
Discharged:
Absconded:
Deceased:

Discharge Diagnosis:	

Discharge Medication:
