# Supplementary material for: Reductions in Inpatient Mortality following Interventions to Improve Emergency Hospital Care in Freetown, Sierra Leone
Source: PLoS One. 2012 Sep 19;7(9):e41458. doi: 10.1371/journal.pone.0041458 (PMC3446969; doi:10.1371/journal.pone.0041458)
Supplement: Figure S2 — Programme for triage and emergency training course. (PDF) [file pone.0041458.s004.pdf]

Ola During Children's Hospital – Triage and Emergency Training October 2009

Day 1

Registration and Welcome (30 mins)
Welcome

Check In and Agenda (30 mins)
Stand in a circle. Each person introduces themselves and describes one thing they are good at. Presentation of the agenda, agreement on start times, conditions for certificates.

What makes a good healthcare professional? (30 mins)
Exercise and discussion involving everyone, emphasising healthcare team roles.

Triage and Emergency: Challenges and Solutions (30 mins)
Brainstorm: what would make a high quality Triage and ER in ODCH? What are the challenges? How can we find solutions?

Child friendly techniques (30 mins)
Introduction to a child friendly approach and some techniques

Triage (1 hr plus 1 hr 30)
- What is Triage? Why do we need it?
- Definition of Emergency/Priority/Routine
- Introducing the processes we will use at ODCH
- ABCD – how to identify emergency and priority patients, what to do with them
- Using of health professional medical records
- In small groups, run through ABCD process and assign example cases to Emergency/Priority/Routine

Equipment and skills workshop 1 (1hr) (All)
3 x 20 minute stations – small groups rotate around the stations.
Station 1: weighing a baby, weighing a child, length/height measurement, identifying malnourished children and alerting others
Station 2: taking a temperature and recording it, working out paracetamol dose, starting tepid sponging
Station 3: taking a pulse, using sats machine, what is abnormal, what to do about it, recording the value

Check out
Stand in a circle. Each person says one thing they learnt today.

TUESDAY 27th October 2009

Check in (30 mins)

Review of Triage (30 mins)
Review of previous day's key messages and skills. Questions and feedback.

An approach to Emergencies (1hr)
Brainstorm on key things needed (e.g. preparation, teamwork, speed)
Teamwork – in theory and in practice e.g. what challenges do the nurses forsee?

Emergency Assessment and Treatment Part 1 (1 hr 30 mins)
Details to follow

Triage and Emergency Scenarios (1 hr 30 mins) (All)
3 x 30 min stations, each with a scenario or two to work through

Equipment and skills workshop 2 (1 hr) (All)
Station 1: Coma – opening airway, recovery position
Station 2: Identifying respiratory distress, using the sats machine, using oxygen
Station 3: Using glucometer, abnormal values, giving glucose

Check out

WEDNESDAY 28th October 2009

Check in (30 mins)

Emergency Assessment and Treatment Part 1 (1 hr 30 mins)
Details to follow

Triage and Emergency Scenarios (1 hr 30 mins) (All)
3 x 30 min stations, each with a scenario or two to work through

Always ready? Managing the Triage and ER (1 hr 30 mins)
A discussion on preparation required, how to monitor equipment usage, maintaining the environment in Triage and ER to maintain high standards (e.g. making sure Drs review patients with a view to discharge from ER appropriately)

Equipment and skills workshop 3 (1 hr) (All)
Station 1: Identifying dehydrated child, emergency fluids, starting ORS/ReSoMal
Station 2: Testing Hb with Haemaccu
Station 3: Giving rectal diazepam in fitting child

Check out

THURSDAY 29th October 2009

Check in (30 mins)

Triage and Emergency Scenarios (3 hrs)
Put people in place and start to run Triage and ER, with support and feedback from trainers

Check out, evaluation and certificates (1 hr)
